# Association of Vitamin D Deficiency with Diabetic Nephropathy in Type 2 Diabetes: A Hospital-Based Cross-Sectional Study

**DOI:** 10.3390/diseases13120405

**Published:** 2025-12-17

**Authors:** Shafia Bashir, Geer Mohammad Ishaq, Insha Mushtaq, Mohammad Ashraf Ganie, Imtiyaz Wani, Muteb Alanazi, Ibrahim Asiri, Arshad Hussain, Kashif Ullah Khan, Sirajudheen Anwar

**Affiliations:** 1 Department of Pharmaceutical Sciences, University of Kashmir, Hazratbal, Srinagar 190006, India; shafia.bashir27@gmail.com (S.B.);; 2 Department of Endocrinology, Sheri-Kashmir Institute of Medical Sciences, Srinagar 190011, India; 3 Department of Nephrology, Sheri-Kashmir Institute of Medical Sciences, Srinagar 190011, India; 4 Department of Clinical Pharmacy, College of Pharmacy, University of Hail, Hail 81442, Saudi Arabia; ms.alanazi@uoh.edu.sa (M.A.);; 5 Department of Quantitative Health Science, Mayo Clinic College of Medicine and Sciences, Scottsdale, AZ 85259, USA; asiri.ibra@gmail.com; 6 Clinical Pharmacy & Pharmacy Practice, Faculty of Pharmacy, University Malaya, Kuala Lumpur 50603, Malaysia; k.khan@um.edu.my; 7 Department of Pharmacology and Toxicology, College of Pharmacy, University of Hail, Hail 81442, Saudi Arabia

**Keywords:** diabetic nephropathy, T2DM, vitamin D, observational study, deficiency

## Abstract

Background/Objective: Diabetic nephropathy (DN), a key microvascular complication of type 2 diabetes (T2DM), drives significant morbidity, mortality, and healthcare costs. Vitamin D deficiency has been linked to renal dysfunction, but its role in DN remains unclear. This study assessed the association between vitamin D status and DN versus T2DM without nephropathy. Methods: This cross-sectional hospital-based study included 399 participants (299 DN, 100 T2DM without nephropathy) at a tertiary endocrine clinic. Demographic, clinical, and biochemical data, including serum 25(OH)D, were collected. Chi-square and Mann–Whitney compared categorical and continuous variables, respectively, and multinomial logistic regression assessed the association between vitamin D status and DN (*p* < 0.05). Results: Patients with DN were older (58.2 ± 7.95 vs. 51.4 ± 9.94 years, *p* < 0.001), had more advanced CKD (stages 2–3b: 84.6% vs. 20.0%, *p* < 0.001), and higher albuminuria (moderate: 80.3% vs. 19.0%; severe: 18.4% vs. 0%, *p* < 0.001). They also showed poorer glycemic control, elevated urea and creatinine, lower serum albumin, dyslipidemia, elevated liver enzymes, and higher uric acid (all *p* < 0.05). Vitamin D deficiency was more prevalent in DN (37.7% vs. 8.0%, *p* < 0.001). Unadjusted multinomial regression indicated that T2DM patients without nephropathy had a 91% lower risk of vitamin D deficiency (RRR 0.09; 95% CI 0.04–0.19, *p* < 0.001) and an 87% lower risk of insufficiency (RRR 0.13; 95% CI 0.05–0.26, *p* < 0.001) compared with DN patients. After adjusting for age, HbA1c, creatinine, duration of diabetes and eGFR, the reduced risk of deficiency remained significant (RRR 0.04; 95% CI 0.01–0.16, *p* < 0.001), while the association with insufficiency was no longer significant (*p* = 0.310). Conclusions: This study shows a significant association between vitamin D deficiency and diabetic nephropathy, though its cross-sectional design precludes causal inference. Reverse causality and residual confounding cannot be excluded. Patients with DN had poorer glycemic control, dyslipidemia, and renal function, along with more frequent vitamin D deficiency. Routine vitamin D monitoring may support early detection and risk stratification in T2DM.

## 1. Introduction

Diabetic nephropathy (DN) is one of the most common microvascular complications of type 2 diabetes mellitus (T2DM) and the leading cause of end-stage renal disease (ESRD) worldwide [1,2]. Approximately 30–40% of individuals with T2DM develop DN during their lifetime, contributing substantially to morbidity, mortality, and healthcare costs [1]. The pathological hallmarks of DN include glomerular basement membrane thickening, mesangial expansion, podocyte injury, and a progressive decline in glomerular filtration rate (GFR), ultimately culminating in kidney failure [2]. Given the multifactorial nature of DN, exploration of additional modifiable factors beyond traditional risks is critical.

Beyond traditional risk factors such as poor glycemic control, hypertension, and dyslipidemia, vitamin D deficiency has emerged as a plausible modifiable risk factor for the onset and progression of DN [3,4]. Vitamin D, classically known for its role in calcium and phosphate metabolism, also exerts pleiotropic effects including modulation of the renin–angiotensin–aldosterone system (RAAS), anti-inflammatory and antifibrotic actions, and protective effects on podocytes [5,6,7]. Hypovitaminosis D is common among individuals with diabetes and has been associated with insulin resistance, albuminuria, and reduced kidney function [8]. Experimental studies demonstrate that activation of the vitamin D receptor (VDR) in podocytes can attenuate glomerular injury and proteinuria, providing a mechanistic basis for its potential protective role in DN [9].

Vitamin D deficiency may contribute to DN through multiple mechanistic pathways. Reduced renal 1α-hydroxylase activity in chronic kidney disease impairs conversion of 25(OH)D to its active form, while urinary loss of vitamin D-binding protein in proteinuria and secondary hyperparathyroidism can further exacerbate deficiency. Activation of the vitamin D receptor (VDR) in podocytes has been shown to attenuate glomerular injury and proteinuria, providing a mechanistic basis for its potential protective role in DN [10]. These mechanisms suggest that low vitamin D status could both reflect and contribute to renal dysfunction in diabetes.

Observational and mechanistic studies have linked low vitamin D levels with adverse renal outcomes in T2DM. Severe 25-hydroxyvitamin D [25(OH)D] deficiency has been associated with higher urine albumin-to-creatinine ratio (UACR) and worse renal prognosis [11], while cohort studies suggest low vitamin D predicts both eGFR decline and progression of albuminuria [12,13]. Yet, despite supportive evidence, the role of vitamin D deficiency in diabetic nephropathy remains uncertain, particularly in real-world settings. To address this gap, we conducted a hospital-based cross-sectional study evaluating the association between vitamin D deficiency and DN in patients with T2DM, with the primary objective of describing how vitamin D status differs between patients with and without nephropathy in routine care.

## 2. Materials and Methods

### 2.1. Study Design and Setting

This was a cross-sectional, hospital-based observational study conducted at the Endocrinology outpatient clinic of a tertiary care public teaching hospital. Eligible patients attending the outpatient clinics were recruited consecutively between April 2022 and March 2025. All participants provided written informed consent prior to enrollment. The study was approved by the Institutional Ethics Committee and conducted in accordance with the Declaration of Helsinki [14].

### 2.2. Study Population

A total of 399 participants were included, comprising 299 patients with diabetic nephropathy (DN) and 100 patients with type 2 diabetes mellitus (T2DM) without nephropathy (control group). DN was defined according to Kidney Disease: Improving Global Outcomes (KDIGO) guidelines as the presence of persistent albuminuria, with UACR ≥ 30 mg/g confirmed in at least two of three consecutive samples, with or without reduced estimated glomerular filtration rate (eGFR < 60 mL/min/1.73 m^2^) [15].

### 2.3. Inclusion and Exclusion Criteria

Eligible participants were adults aged ≥ 18 years with a confirmed diagnosis of T2DM based on American Diabetes Association (ADA) criteria [16]. Exclusion criteria included type 1 diabetes, gestational diabetes, or secondary causes of nephropathy such as glomerulonephritis, obstructive uropathy, or polycystic kidney disease. Patients with acute kidney injury, chronic liver disease, or systemic infections were also excluded. To minimize confounding, individuals who had received vitamin D supplementation or active vitamin D analogs within the preceding three months were not enrolled.

### 2.4. Clinical and Demographic Assessment

Clinical and demographic information was collected using a structured case record form and verified against medical records. Variables recorded included gender, age (in years), residence status (rural or urban), and body mass index (BMI). Lifestyle factors such as exercise frequency (daily, rare, or never) and smoking status (current, past, or never) were documented. Socioeconomic details included income source (regular vs. no regular income). Family history of diabetes was also noted. Clinical details comprised diagnosis type (DN and T2DM without nephropathy), duration of diabetes (years since diagnosis), and the presence of comorbidities such as hypertension, anemia, hypothyroidism, dyslipidemia, etc.

### 2.5. Laboratory Measurements

Laboratory results were obtained directly from patient medical records during face-to-face interviews. Glycemic status was assessed through fasting blood glucose (FBS) and glycated hemoglobin (HbA1c). Blood pressure was documented as systolic (SBP) and diastolic (DBP) values. Renal function was evaluated by serum urea, serum creatinine, estimated glomerular filtration rate (eGFR, calculated using the CKD-EPI equation [16]), and CKD staging. Spot urine albumin and creatinine were measured to calculate the albumin-to-creatinine ratio (ACR). Additional biochemical variables included alkaline phosphatase (ALP), total protein, serum glutamic oxaloacetic transaminase (SGOT), serum glutamic pyruvic transaminase (SGPT), serum albumin, bilirubin, calcium, phosphorus, sodium (Na), potassium (K), and uric acid. Hematological evaluation included hemoglobin (Hb). Lipid profile assessment comprised total cholesterol, high-density lipoprotein (HDL), low-density lipoprotein (LDL), and triglycerides (TG). Serum 25-hydroxyvitamin D (25(OH)D) had been measured using a chemiluminescence immunoassay. Vitamin D status was categorized according to Endocrine Society guidelines as deficient (<20 ng/mL), insufficient (20–29.9 ng/mL), or sufficient (≥30 ng/mL) [17]. UACR was calculated from spot urine albumin and creatinine measurements, with values classified according to KDIGO guidelines as normal (<30 mg/g), moderately increased (30–300 mg/g), or severely increased (>300 mg/g) [15].

### 2.6. Statistical Analysis

Continuous variables were expressed as mean ± standard deviation (SD), and categorical variables as frequencies and percentages. Between-group differences were evaluated using Mann–Whitney (non-parametric test) for continuous data and Chi-square test for categorical data. The independent association between vitamin D status and DN was assessed using multinomial logistic regression models, with a priori adjustment for key clinical confounders (age, HbA1c, serum creatinine, eGFR, and duration of diabetes), given their established roles in diabetic nephropathy and potential relationships with vitamin D status. Results were expressed as relative risk ratios (RRRs) with 95% confidence intervals (CIs). Missing data were assessed for extent and patterns. Variables with <15% missingness were imputed using multiple imputation, assuming data were missing at random. Variables with excessive missingness were not imputed to avoid bias. All statistical analyses were performed in STATA 18, and a *p*-value < 0.05 was considered statistically significant.

## 3. Results

It should provide a concise and precise description of the experimental results, their interpretation, as well as the experimental conclusions that can be drawn.

### 3.1. Demographic and Clinical Characteristics

A total of 399 participants were included in the analysis, comprising 299 with diabetic nephropathy and 100 with type 2 diabetes mellitus without nephropathy. The demographic and clinical characteristics of the study population are summarized in Table 1.

Patients with DN were significantly older than those without nephropathy (123 (41.1%) vs. 18 (18%); *p* < 0.001). Gender distribution was comparable between groups, with females accounting for roughly two-thirds of participants in both cohorts (*p* = 0.82). Body mass index differed significantly, as low or normal BMI was more frequent in the T2DM group compared with DN (70.0% vs. 58.9%; *p* = 0.047). Residence status, smoking behavior, and income source were similar across the two groups (all *p* > 0.05).

Lifestyle characteristics also showed modest differences. Daily exercise was reported by 55.0% of participants with T2DM and 43.5% of those with DN, although this difference did not reach statistical significance (*p* = 0.082). Other exercise frequencies, as well as smoking status, showed no meaningful variation between groups.

Marked contrasts were observed in kidney disease staging. The majority of participants with T2DM were in CKD stage 1 (80.0%), while DN patients were more frequently distributed across stages 2 (47.8%), 3a (23.4%), and 3b (13.4%) (*p* < 0.001).

A similar pattern was seen for albumin-to-creatinine ratio categories. Most DN patients were in moderate microalbuminuria (80.3%) or severe macroalbuminuria (18.4%), whereas nearly half of T2DM participants were in the normal ACR range (49.0%), with a substantial proportion lacking ACR data (32.0%) (*p* < 0.001).

Family history of T2DM was more common among participants with DN than those without nephropathy (53.7% vs. 40.0%, *p* = 0.018). Disease duration was strongly associated with nephropathy status. Among individuals with shorter diabetes duration (≤5 years), 65.0% were in the T2DM without nephropathy group compared with only 19.7% of DN patients. Conversely, long-standing diabetes (>5 years) was observed in 80.3% of DN patients compared with 35.0% of those without nephropathy (*p* < 0.001).

### 3.2. Biochemical Characteristics

The biochemical characteristics of the study population are presented in Table 2. Continuous biochemical variables were not normally distributed in at least one group; therefore, data are presented as median [interquartile range], and between-group comparisons were performed using the Mann–Whitney U test.

Patients with DN were significantly older than those without nephropathy (median age 58 (51–63)vs. 51 (46–57) years; *p* < 0.001). Glycemic parameters, including HbA1c and fasting blood sugar, were also higher in the DN group (HbA1c 8.2 (7.5–9.0%) vs. 7.3 (6.8–7.7%), *p* < 0.001; FBS 149 (135–162) mg/dL vs. 142 (130–155) mg/dL, *p* = 0.0005).

Renal function markers were worse in the DN group, with higher urea (31 (24–38) mg/dL vs. 22 (18–27) mg/dL; *p* < 0.001) and creatinine levels (1.35 (1.0–1.7) mg/dL vs. 0.82 (0.7–0.9) mg/dL; *p* < 0.001). Serum albumin was lower in DN patients (4.0 (3.7–4.3) g/dL vs. 4.48 (4.3–4.6) g/dL; *p* < 0.001), whereas hemoglobin levels were comparable between groups (*p* = 0.466).

Lipid profile analysis showed that HDL was lower (42 (37–47) mg/dL vs. 48 (44–52) mg/dL; *p* < 0.001), while LDL levels did not differ significantly (90 (74–110) mg/dL vs. 86 (77–98) mg/dL; *p* = 0.431). Triglycerides and TG/HDL ratio were higher in DN patients (176 (120–220) mg/dL vs. 134 (100–170) mg/dL, *p* < 0.001; TG/HDL 4.4 (2.9–6.0) vs. 2.9 (2.0–3.5), *p* < 0.001).

Liver function tests, including SGPT and SGOT, were elevated in DN (SGPT 30 (22–36) vs. 18 (15–22) U/L, *p* < 0.001; SGOT 25 (19–31) vs. 21 (16–25) U/L, *p* = 0.013). ALP levels, sodium, and potassium showed no clinically meaningful differences. Uric acid was higher in DN patients (5.7 (4.8–6.6) mg/dL vs. 4.4 (3.8–5.0) mg/dL; *p* = 0.0061).

### 3.3. Prevalence of Vitamin D Deficiency in DN and T2DM Without DN

**Figure 1:**
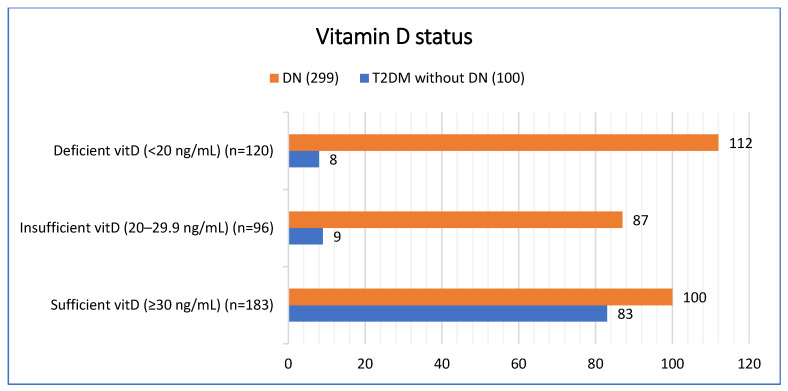
Vitamin D status based on patient groups.

The prevalence of vitamin D deficiency and insufficiency differed significantly between groups (*p* < 0.001). Among patients with diabetic nephropathy, 37.4% (*n* = 112) were vitamin D deficient and 29.1% (*n* = 87) were insufficient, compared with 8.0% (*n* = 8) and 9.0% (*n* = 9), respectively, in patients with T2DM without DN. Conversely, vitamin D sufficiency was more common in the T2DM without DN group 83.0% (*n* = 83) compared with the DN group 33.4% (*n* = 100) [Figure 1].

In the subgroup analysis, Vitamin D status did not differ significantly between males and females (χ^2^(2) = 0.68, *p* = 0.71). Among females (*n* = 271), 78 (28.78%) were deficient, 66 (24.35%) were insufficient, and 127 (46.86%) were sufficient. Among males (*n* = 128), 42 (32.81%) were deficient, 30 (23.44%) were insufficient, and 56 (43.75%) were sufficient. Vitamin D status was significantly associated with age (χ^2^(2) = 9.52, *p* = 0.009). Among adults <60 years (*n* = 258), 76 (29.46%) were deficient, 51 (19.77%) were insufficient, and 131 (50.78%) were sufficient. Among elderly ≥60 years (*n* = 141), 44 (31.21%) were deficient, 45 (31.91%) were insufficient, and 52 (36.88%) were sufficient. No significant differences were observed across BMI groups (χ^2^(2) = 3.04, *p* = 0.218). In the normal/low BMI group (*n* = 246), 76 (30.89%) were deficient, 65 (26.42%) insufficient, and 105 (42.68%) sufficient. In the obese/overweight group (*n* = 153), 44 (28.76%) were deficient, 31 (20.26%) insufficient, and 74 (50.98%) sufficient. Vitamin D status was significantly associated with disease duration (χ^2^(2) = 8.66, *p* = 0.013). Among patients with shorter duration (≤5 years, *n* = 124), 35 (28.23%) were deficient, 20 (16.13%) were insufficient, and 68 (55.65%) were sufficient. Among patients with longer duration (>5 years, *n* = 275), 85 (30.91%) were deficient, 76 (27.64%) were insufficient, and 114 (41.45%) were sufficient [Table 3].

### 3.4. Association Between Vitamin D Deficiency with DN and T2DM Without DN

We investigated the association between patient group (diabetic nephropathy vs. type 2 diabetes mellitus without nephropathy) and vitamin D status using multinomial logistic regression. Vitamin D sufficiency (30–100 ng/mL) was used as the reference outcome, and DN served as the reference group for patient group comparisons.

In unadjusted analyses, compared with DN patients, the relative risk of vitamin D deficiency was substantially lower among T2DM patients without nephropathy (RRR = 0.09, 95% CI: 0.04–0.19, *p* < 0.001), corresponding to a 91% lower risk. Similarly, the relative risk of vitamin D insufficiency was markedly reduced in this group (RRR = 0.13, 95% CI: 0.06–0.26, *p* < 0.001), reflecting an 87% lower risk. These findings indicate that vitamin D deficiency and insufficiency are significantly more prevalent among individuals with DN compared with those with T2DM without nephropathy [Table 4].

After adjusting for age, HbA1c, creatinine, eGFR, and duration of diabetes, variables selected a priori because longer diabetes duration and worse metabolic control are central determinants of both DN and vitamin D status, the association with deficiency remained strong and statistically significant (RRR = 0.041; 95% CI: 0.01–0.16; *p* < 0.001), whereas the lower relative risk of insufficiency (RRR = 0.56; 95% CI: 0.18–1.71) did not reach significance (*p* = 0.310). The adjusted model was significant (LR χ^2^(10) = 90.90, *p* < 0.001), showing patient group as a key determinant of vitamin D status. The modest variance explained (pseudo-R^2^ = 0.11) indicates these findings are exploratory rather than predictive. Given that data on hypertension was insufficiently documented, hypertension was excluded from the primary adjusted model. Therefore, the possibility of residual confounding by hypertension and other unmeasured variables must be acknowledged when interpreting the observed association between vitamin D status and DN.

## 4. Discussion

This study demonstrated a markedly higher prevalence of vitamin D deficiency and insufficiency among patients with diabetic nephropathy (DN) compared with those with T2DM without nephropathy. Nearly two-thirds (67%) of individuals with DN had suboptimal vitamin D status, whereas vitamin D sufficiency predominated in the non-DN group. These findings are consistent with prior evidence reporting lower serum 25-hydroxyvitamin D [25(OH)D] concentrations among patients with diabetic microvascular complications [18], with individuals with nephropathy exhibiting higher rates of deficiency than those without nephropathy [19]. Collectively, these observations reinforce the growing body of literature suggesting a potential link between vitamin D status and the development or progression of DN.

In subgroup analyses, vitamin D status did not differ significantly by sex or BMI, suggesting that renal involvement may be a more important determinant of vitamin D levels than anthropometric characteristics. This aligns with findings from Hong et al. (2021), who reported that vitamin D deficiency in Korean patients with T2DM was more strongly associated with the presence of nephropathy than with sex or body size [20]. In contrast, increasing age and longer diabetes duration were associated with lower vitamin D sufficiency in our cohort. These observations are consistent with prior evidence of age-related declines in cutaneous vitamin D synthesis, including a ~13% reduction in vitamin D_3_ production per decade following standardized ultraviolet exposure (Chalcraft et al., 2020) and a >50% reduction in skin 7-dehydrocholesterol between ages 20 and 80, as noted in the international consensus by Giustina et al. (2022) [21,22]. Additionally, longstanding diabetes and cumulative metabolic burden may contribute to hypovitaminosis D through mechanisms involving insulin resistance and β-cell dysfunction (Chiu et al., 2004) [23]. These findings suggest that age-related physiological changes and chronic diabetes exposure, rather than sex or BMI, are key determinants of vitamin D status in patients with T2DM, highlighting the need for closer monitoring of older adults and those with longer disease duration who may be at greater risk of deficiency and DN progression.

Multinomial regression demonstrated a significant association between vitamin D deficiency and DN, which remained robust after adjustment for age, HbA1c, creatinine, diabetes duration, and eGFR. However, the cross-sectional design limits causal interpretation, and reverse causality or residual confounding cannot be excluded. These results are further supported by a recent meta-analysis indicating that low vitamin D status is associated with both increased risk and greater severity of DN [24]. Although the association is strong, the relationship may be bidirectional: DN can reduce renal 1α-hydroxylase activity, impair conversion of 25(OH)D to its active form, and increase urinary loss of vitamin D-binding protein, with secondary hyperparathyroidism further exacerbating deficiency in chronic kidney disease [25]. Conversely, vitamin D may influence DN pathogenesis through activation of the vitamin D receptor (VDR), which suppresses renin–angiotensin–aldosterone system activity, reduces podocyte injury, and limits glomerular inflammation and fibrosis [5]. Additionally, potential seasonal variation in sunlight exposure was not fully accounted for, as the timing of blood draws differed between DN and non-DN groups, which may have influenced vitamin D levels and introduced bias. Given this interplay, the direction of causality cannot be determined from these observational data, and findings should be interpreted as evidence of association rather than causation.

Interestingly, the association between vitamin D insufficiency and DN was attenuated after adjustment for confounders, whereas the relationship with frank deficiency persisted. This suggests that severe deficiency may be more directly linked to renal injury, while moderate insufficiency may overlap with other risk factors such as glycemic control and reduced kidney function, consistent with prior studies showing stronger associations for deficiency than insufficiency [26,27,28].

The strengths of this study include a relatively large hospital-based sample, standardized biochemical and vitamin D assessments, subgroup analyses, and multivariable adjustment for key clinical covariates. However, several limitations warrant consideration. The cross-sectional, observational design precludes causal inference, and the lack of longitudinal follow-up prevents assessment of whether vitamin D deficiency predicts progression of DN. Vitamin D was measured only once, and potential confounders such as diet, supplementation, sunlight exposure, and other biomarkers (e.g., parathyroid hormone, FGF-23) were not assessed. Furthermore, the single-center design and group imbalance (299 DN vs. 100 controls) may introduce selection bias and limit generalizability.

The findings also invite future prospective studies to investigate whether vitamin D deficiency predicts longitudinal changes in renal outcomes among patients with T2DM. Randomized controlled trials stratified by baseline vitamin D status are warranted to determine whether targeted supplementation can prevent or slow the DN progression. Such trials should incorporate relevant biomarkers, including parathyroid hormone, FGF-23, UACR, eGFR, and lipid parameters, to better characterize risk profiles and identify patients most likely to benefit from intervention. Integrating vitamin D measurements into multi-biomarker risk models may enhance personalized risk stratification and clinical decision-making.

## 5. Conclusions

This study demonstrates that vitamin D deficiency is significantly associated with diabetic nephropathy, independent of other metabolic abnormalities. However, given the cross-sectional design, causality cannot be inferred, and the possibility of reverse causality and residual confounding remains. DN patients exhibited worse glycemic control, dyslipidemia, and renal dysfunction alongside higher rates of vitamin D deficiency. These findings support the potential value of routinely assessing vitamin D status in patients with T2DM, particularly those with longer disease duration or declining renal function. Identifying and addressing vitamin D deficiency may aid early risk stratification and guide individualized monitoring for DN progression.

## Figures and Tables

**Table 1:** Demographic and Clinical Characteristics by DN and T2DM without DN.

Variable	Category	Total (*n* = 399)	Diabetic Nephropathy (*n* = 299)	T2DM Without Nephropathy (*n* = 100)	*p*-Value
Age	Adults	258 (64.7)	176 (58.9)	82 (82.0)	<0.001
	Elderly	141 (35.3)	123 (41.1)	18 (18.0)	
Gender	Female	271 (67.9)	204 (68.2)	67 (67.0)	0.820
	Male	128 (32.1)	95 (31.8)	33 (33.0)	
BMI	Low/Normal	246 (61.7)	176 (58.9)	70 (70.0)	0.047
	Obese/Overweight	153 (38.3)	123 (41.1)	30 (30.0)	
Residence Status	Rural	244 (61.2)	183 (61.6)	61 (61.0)	0.913
	Urban	153 (38.4)	114 (38.4)	39 (39.0)	
Exercise Status	Daily	183 (46.5)	128 (43.5)	55 (55.0)	0.082
	Never	79 (20.0)	59 (20.1)	20 (20.0)	
	Rarely	132 (33.5)	107 (36.4)	25 (25.0)	
Smoking Status	Current smoker	43 (10.8)	35 (11.7)	8 (8.0)	0.356
	No	335 (84.0)	247 (82.6)	88 (88.0)	
	Past smoker	21 (5.3)	17 (5.7)	4 (4.0)	
Income Source	No regular income	255 (63.9)	189 (63.2)	66 (66.0)	0.615
	Regular income	144 (36.1)	110 (36.8)	34 (34.0)	
CKD Stage	1	90 (22.6)	10 (3.3)	80 (80.0)	<0.001
	2	163 (40.9)	143 (47.8)	20 (20.0)	
	3a	70 (17.5)	70 (23.4)	0 (0.0)	
	3b	40 (10.0)	40 (13.4)	0 (0.0)	
	4	31 (7.8)	31 (10.4)	0 (0.0)	
	5	5 (1.3)	5 (1.7)	0 (0.0)	
ACR Stage	Missing	36 (9.0)	4 (1.3)	32 (32.0)	<0.001
	Normal (<30 mg/g)	49 (12.3)	0 (0.0)	49 (49.0)	
	Mod. Microalbuminuria (30–299 mg/g)	259 (64.9)	240 (80.3)	19 (19.0)	
	Severe Macroalbuminuria (≥300 mg/g)	55 (13.8)	55 (18.4)	0 (0.0)	
Duration of T2DM	Short (≤5 years)	124 (31.1)	59 (19.7)	65 (65.0)	<0.001
	Long (>5 years)	275 (68.9)	240 (80.3)	35 (35.0)	
Family history of T2DM	No	199 (49.9)	139 (46.4)	60 (60.0)	0.018
	Yes	200 (50.1)	160 (53.6)	40 (40.0)	

Data are presented as *n* (%). *p*-values are derived from Pearson chi-square tests comparing diabetic nephropathy vs. T2DM.

**Table 2:** Biochemical Characteristics by DN and T2DM without DN.

Variable	Total (*n* = 399)	DN (*n* = 299)	T2DM Without DN (*n* = 100)	*p*-Value (Mann–Whitney U)
Age (years)	56 [50–63]	58 [51–63]	51 [46–57]	<0.001
HbA1c (%)	7.9 [7.3–8.9]	8.2 [7.5–9.0]	7.3 [6.8–7.7]	<0.001
FBS (mg/dL)	146 [134–161]	149 [135–162]	142 [130–155]	0.0005
Urea (mg/dL)	27 [21–34]	31 [24–38]	22 [18–27]	<0.001
Creatinine (mg/dL)	1.2 [0.9–1.6]	1.35 [1.0–1.7]	0.82 [0.7–0.9]	<0.001
Serum Albumin (g/dL)	4.1 [3.8–4.4]	4.0 [3.7–4.3]	4.48 [4.3–4.6]	<0.001
Hemoglobin (g/dL)	12.6 [11.5–13.7]	12.6 [11.5–13.7]	12.7 [11.8–13.6]	0.466
HDL (mg/dL)	44 [39–48]	42 [37–47]	48 [44–52]	<0.001
LDL (mg/dL)	89 [75–105]	90 [74–110]	86 [77–98]	0.431
Triglycerides (mg/dL)	162 [110–210]	176 [120–220]	134 [100–170]	<0.001
SGPT (U/L)	25 [18–32]	30 [22–36]	18 [15–22]	<0.001
SGOT (U/L)	24 [18–29]	25 [19–31]	21 [16–25]	0.0128
ALP (U/L)	82 [65–102]	85 [66–105]	75 [62–90]	0.164
Na (mmol/L)	138 [136–141]	138 [136–141]	140 [138–141]	0.0848
K (mmol/L)	4.1 [3.7–4.5]	4.0 [3.6–4.4]	4.1 [3.9–4.5]	0.0393
Uric Acid (mg/dL)	5.2 [4.2–6.2]	5.7 [4.8–6.6]	4.4 [3.8–5.0]	0.0061
TG/HDL ratio	4.1 [2.7–5.6]	4.4 [2.9–6.0]	2.9 [2.0–3.5]	<0.001

Note: Data are presented as median [interquartile range]. *p* values were derived from the Mann–Whitney U test comparing DN vs. T2DM without nephropathy.

**Table 3:** Vitamin D Status across different subgroups.

Subgroups	*n* (399)	Vitamin D Deficient (*n*, %)	Vitamin D Insufficient *n*, %)	Vitamin D Sufficient (*n*, %)	*p* Value
**Gender**					0.710
Female	271	78 (28.78%)	66 (24.35%)	127 (46.86%)	
Male	128	42 (32.81%)	30 (23.44%)	56 (43.75%)	
**Age Category**					0.009
Adults	258	76 (29.46%)	51 (19.77%)	131 (50.78%)	
Elderly	141	44 (31.21%)	45 (31.91%)	52 (36.88%)	
**BMI Category**					0.218
Low/Normal	246	76 (30.89%)	65 (26.42%)	105 (42.68%)	
Overweight/Obese	153	44 (28.76%)	31 (20.26%)	78 (50.98%)	
**Disease Duration**					0.013
(≤5 years DM)	124	35 (28.23%)	20 (16.13%)	69 (55.65%)	
(>5 years DM)	275	85 (30.91%)	76 (27.64%)	114 (41.45%)	

**Table 4:** Association of Vitamin D status with DN and T2DM without DN.

Vitamin D Category	Relative Risk Ratio (Unadjusted RRR 95% CI)	*p*-Value	Relative Risk Ratio (Adjusted * RRR 95% CI)	*p*-Value
Deficient (<20 ng/mL)	0.09 (0.04–0.19)	<0.001	0.04 (0.01–0.16)	<0.001
Insufficient (20–29.9 ng/mL)	0.13 (0.06–0.26)	<0.001	0.56 (0.18–1.71)	=0.310
Sufficient (≥30 ng/mL)	Reference	–	Reference	–

* Adjusted for age, HbA1c, creatinine, eGFR, and disease duration.

## Data Availability

The datasets generated and/or analyzed during the current study are not publicly available due to institutional and ethical restrictions.

## Abbreviations

The following abbreviations are used in this manuscript:

Abbreviation	Full Form
25(OH)D	25-Hydroxyvitamin D
ADA	American Diabetes Association
ACR	Albumin-to-Creatinine Ratio
ALP	Alkaline Phosphatase
ALT/SGPT	Serum Glutamic Pyruvic Transaminase
AST/SGOT	Serum Glutamic Oxaloacetic Transaminase
BMI	Body Mass Index
BP	Blood Pressure
CKD	Chronic Kidney Disease
CKD-EPI	Chronic Kidney Disease Epidemiology Collaboration
DN	Diabetic Nephropathy
DM	Diabetes Mellitus
eGFR	Estimated Glomerular Filtration Rate
ESRD	End-Stage Renal Disease
FBS	Fasting Blood Sugar
HbA1c	Glycated Hemoglobin
HDL	High-Density Lipoprotein
KDIGO	Kidney Disease: Improving Global Outcomes
LDL	Low-Density Lipoprotein
Na	Sodium
K	Potassium
PTH	Parathyroid Hormone
RAAS	Renin–Angiotensin–Aldosterone System
RRR	Relative Risk Ratio
SD	Standard Deviation
STATA 18	Statistical Analysis Software
T2DM	Type 2 Diabetes Mellitus
TG	Triglycerides
UACR	Urine Albumin-to-Creatinine Ratio
VDR	Vitamin D Receptor
